# Genetic Diagnostic Methods for Inherited Eye Diseases

**DOI:** 10.4103/0974-9233.75881

**Published:** 2011

**Authors:** Luis A. R. Gabriel, Elias I. Traboulsi

**Affiliations:** Department of Pediatric Ophthalmology and Strabismus and the Center for Genetic Eye Diseases, Cole Eye Institute, Cleveland Clinic, Cleveland, USA

**Keywords:** Diagnosis, Genetics, Karyotype, Laboratory, Mutation

## Abstract

Accurate molecular diagnosis of genetic eye diseases has proven to be of great importance because of the prognostic and therapeutic value of an accurate ascertainment of the underlying genetic mutation. Efforts continue in diagnostic laboratories to develop strategies that allow the discovery of responsible gene/mutations in the individual patient using the least number of assays and economizing on the expenses and time involved in the process. Once the ophthalmologist has made the best possible clinical diagnosis, blood samples are obtained for genetic testing. In this paper we will review the basic laboratory methods utilized to identify the chromosomal or mutational etiology of genetic diseases that affect the eye.

## INTRODUCTION

Accurate molecular diagnosis of genetic eye diseases has proven to be of great importance since the discovery that clinically similar diseases such as retinitis pigmentosa, Leber congenital amaurosis (LCA), or Bardet-Biedl syndrome (BBS) could be in fact caused by mutations in different genes (genetic heterogeneity), and that clinically dissimilar diseases such as some of the corneal dystrophies result from different mutations in the same gene (phenotypic heterogeneity). The clinical significance of the ability to make these types of differentiations resides in the diagnostic, prognostic, and therapeutic values of an accurate ascertainment of the underlying genetic mutation. Obviously, if a possibility of treatment (even on a research basis) is based on gene replacement or on manipulation of the pathophysiological mechanisms that underlie the disease, an accurate molecular genetics diagnosis is paramount. A recent example of this paradigm is the advent of viral-mediated gene therapy for one subtype of patients with LCA who have mutations in *RPE65*, while other LCA patients who have mutations in more than 15 other genes are still awaiting therapeutic breakthroughs.^1^–^3^

An educated and most likely clinical diagnosis is essential in guiding molecular testing and ensuring the highest possible yield of expensive and complicated laboratory work. While clinical diagnosis and choosing the appropriate gene to test is relatively straightforward for example in patients with classic vitelliform *Best disease*, it is much more difficult in an isolated case of retinitis pigmentosa where testing for a number of genes may only disclose the underlying mutation in a relatively small percentage of cases. Efforts continue in diagnostic laboratories to develop strategies that allow the discovery of responsible gene/mutations in the individual patient using the least number of assays and economizing on the expenses and time involved in the process. Pooling of samples, the utilization of automated sequencers and microarray chips, and the testing for more common mutations before complete gene sequencing are only a few of the approaches that are currently utilized.

## IN THE CLINIC

Once the ophthalmologist has made the best possible clinical diagnosis utilizing the information provided by family history, symptomatology, physical examination, electrodiagnostic testing, manual Goldmann perimetry, and imaging techniques, such as ocular coherence tomography (OCT), the patient is informed and consented for the obtaining of a sample that would provide DNA for analysis. This is usually a peripheral blood sample, although sputum or mouth swabs could be an acceptable source for some assays. It is important that DNA testing be offered within the context of appropriate genetic counseling. Some physicians are confident in their abilities to provide such services while others choose to refer their patients to specialized centers where this work-up can be performed.

Laboratories that provide genetic testing for a particular disease can be identified using for example http://www.genetests.org, a website that allows the user to look for availability around the world of genetic testing for the disease in query, and for a list of fee-for-service and of research laboratories. Blood samples are then obtained and contact is made with the laboratory for handling of samples and necessary clinical information. The list of disorders that could be tested for changes constantly as new laboratories offer tests and new genes are discovered. It is important to check for the most recent lists on the appropriate websites.

While some laboratories such as the Carver Laboratory (http://www.carverlab.org) in Iowa City, USA, provide a non-for-profit service and accept samples for a large variety of diseases, other laboratories have pooled their resources and formed a consortium in which samples are handled via a central service/program at the National Eye Institute (NEI) in Bethesda, USA. This latter program, eyeGene (http://www.nei.nih.gov/resources/eyegene.asp), provides not only mutation analysis for the individual patient, but will keep DNA samples and make them available to researchers. Several commercial laboratories such as GeneDx (http://www.genedx.com) in Washington, D.C., USA, provide excellent services and a wide range of diagnostic capabilities. The authors are not very familiar with European laboratories.

## GENETIC TESTING AND CLIA-CERTIFIED LABORATORIES

Validated laboratory procedures specified by the Clinical Laboratory Improvement Amendment (CLIA) must be followed by every laboratory that offers clinical genetic testing in the USA. These are laboratories that provide physicians and patients with official interpretative reports of their findings. The guidelines for the performance of tests and the functioning of the laboratories have been developed in order to assure excellent and reliable quality based on two essential components: (1) standardization of methods; and (2) inter-laboratory comparison of test results. The standardized methods for performance of the most trivial clinical molecular tests are provided by the Clinical and Laboratory Standards Institute CLSI (http://www.clsi.org). The inter-laboratory comparison of test performance is obtained from the College of American Pathologists CAP (http://www.cap.org).

## TECHNIQUES FOR IDENTIFYING UNDERLYING GENETIC ETIOLOGY OF EYE DISORDERS

In this section we will briefly explain the laboratory techniques that are utilized to analyze chromosomes and genes for abnormalities. This review is not means to be exhaustive but will give the reader some information on the subject.

### Cytogenetic tests

Cytogenetic tests allow an analysis of the number and morphology of chromosomes and the detection of chromosomal duplications, deletions, insertions, or translocations.

#### Karyotype

The karyotype was created by the Ukranian botanist Grigorii Andreevich Levitsky in 1924.^4^,^5^ The chromosomes are analyzed after a photomicrograph is taken using a camera mounted on the light microscope and analyzed using a computer with proper software. Chromosomes are rearranged in a predetermined fashion called karyogram or idiogram (in pairs and by the same centromere position in each pair with the short arm “p” (from the french word *petit*) on top and numbered from 1 to 23. Cells (usually lymphocytes from peripheral blood) are grown *in vitro* and are treated with special culture techniques since these cells do not undergo mitosis physiologically. Karyotypes are usually obtained in patients with multiple congenital malformations and those suspected of having known syndromes such as Down or Patau. They are also performed on amniocytes obtained for prenatal screening of fetuses for chromosomal abnormalities. The most common prenatal analysis specimen is 20 to 30 mL of amniotic fluid drawn by amniocentesis usually between 16 and 18 weeks of gestation. The risk of fetal death due to this procedure is around 0.5%. If an earlier diagnosis is necessary, a transabdominal or transvaginal chorionic villus sample can be obtained between 10 and 14 weeks of gestation; however the risk of fetal death related to this approach is twice as high: 1%. If a quick karyotype result is needed during a more advanced gestation (higher than 20 weeks), then a cordocentesis, which carries a chance of 2% to 5% of fetal death, can be performed.^6^–^11^ Blood, amniotic fluid and chorionic villi should be kept at room temperature and be transported to the laboratory without delays. The specimens are then processed and the chromosome spreads on slides are prepared. Giemsa or Wright stains, which are positively charged dyes that attach to the negatively charged DNA, are generally used. The Giemsa method is used to dye DNA phosphate groups providing a specific pattern of 300-400 lightly or darkly stained bands called G-bands. The banding pattern is unique for each chromosome pair, facilitating their identification. Additional Q-, C-, or R-banding can be obtained in special cases. The Q-banding, performed with quinacrine fluorescence staining is useful for rapid Y chromosome identification, because the edge of its q arm is composed of heterochromatin rendering the most fluorescent region in human metaphase cells. C-banding, named after constitutive or centromere banding, is employed to evaluate constitutive heterochromatin or chromosome dicentricity. Normally, the centromere should appear as a dark spot on a pale chromosome. R-banding, named after reverse banding, dyes the same regions that the G-banding does, though reversing the dark and light regions. Since the telomeres are light stained in the G-banding, this approach is useful for telomere evaluation because the telomeres are many times too small to be evaluated under light staining.^12^–^15^

Karyotype analysis starts under the light microscope with chromosome counting defined by the total number of centromeres, which should be 46 in the diploid cells. Since artifacts can occur, 15 to 20 cells must be analyzed. In mosaic cases, 25 to 50 cells must be evaluated. The identification of each chromosome is based on the size, centromere position, and banding pattern. Commonly, subtle deletions may not be detected using this type of analysis. Therefore, prometaphase harvest, when the chromosomes are less condensed must be carried out to try to see this smaller alterations.^12^–^15^

Syndromes like Turner (45,X), Klinefelter (47,XXY), Edwards (Trisomy 18), Down (Trisomy 21), and Patau (Trisomy 13) can be diagnosed by this method, and so can some types of cancers like the chronic myelogenous leukemia associated with translocation between chromosomes 9 and 22, t(9;22)(q34;q11), also known as Philadelphia chromosome.^16^ Down syndrome occurs with increasing frequency with advancing maternal age. 92.5% of patients have 47 chromosomes with a trisomy of chromosome 21. Less than 3% present a milder phenotype due to mosaicism, with two cell lines (47,XX,+21/46,XX or 47,XY,+21/46,XY), and approximately 5% have 46 chromosomes with the third chromosome 21 (essentially containing bands 21q22.12 – 21q22.3) as part of a translocation. In this last case, the parents must undergo karyotype too in order to evaluate parental translocations, and therefore, the chance of another birth with the same condition. Ocular manifestations include slanted palpebral fissures and a higher incidence of strabismus, refractive errors, cataract (postulated that its augmented occurrence may be due to the alpha A-crystallin gene located at the 21) and keratoconus.^17^–^19^

#### FISH analysis

Fluorescence *in situ* hybridization (FISH) involves a combination of cytogenetic and molecular techniques. As opposed to the standard karyotype in which a dye is used to bind the chromosome, a fluorescent DNA probe is used. The types of probes are: (1) chromosome painting probes that represent a pool of many DNA fragments directed against the entire length of a chromosome making them totally fluorescent; these are useful when there is a supranumerary chromosome in order to try to discover the origin of the duplicated region; (2) repeat sequence probes which can be directed against centromeres for chromosome counting, or against telomeres to check the telomere presence; and (3) unique sequence probes to locate a particular gene or DNA region. With regard to the technique, chromosomes can be arrested either in metaphase or interphase. One advantage of the interphase FISH over the metaphase FISH is that when the chromosome affected length is shorter than 500-1000 kb, its identification on metaphase chromosomes becomes hard to assess, thus in cases like this the interphase FISH could be an alternative. For metaphase, culture is conducted in the same way as to the common karyotype, but for interphase capture culture is not required. After fixation, the double-stranded DNA on the slides is denaturated and transformed to single-stranded DNA in order to be hybridized to fluorescent-labeled probes at an annealing temperature. The unbound probes are washed away and a counterstaining using another fluorochrome is performed. This technique is particularly valuable for microdeletions (500-650 kb), where a probe directed against the probable deleted sequence will not hybridize in the case of a correct diagnostic hypothesis, but, in contrast, will hybridize if the patient does not carry the presumed microdeletion. Therefore, the same alleles in both chromosomes can be compared regarding their fluorescence, in a way that if both show fluorescence, there is no microdeletion at all, but if only one shows fluorescence, then the microdeletion is confirmed. Cases like this may occur in the so-called haploinsufficiency model in which a mutation or a deletion in one of the alleles generates a loss of function of the affected region that is not fully compensated by the other allele. One example of this kind of disease is the Williams-Beuren Syndrome. In cases where duplication is expected, then three fluorescent regions should be observed: two from the normal alleles and one from the abnormal one. A positive control, ideally with a different color, and against another region of the same chromosome arm is applied. A total of 20 cells must show the same result in order to give the diagnosis. Some examples are the microdeletion of about 26 genes on 7q11.23 in Williams-Beuren Syndrome patients, and the Prader-Willi and the Angelman syndrome who have del(15)(q11.2q11.2). Patients with the WAGR (Wilms’ tumor, Aniridia, Genito-urinary malformations and Retardation) syndrome have deletions of 11p13. Standard fluorescence microscopes allow the visualization of only three colors; however a coloring method and image capturing using a computer-assisted system make it possible to attribute different colors to all of the different 24 types of chromosomes.^20^–^24^

#### SKY test

The spectral karyotype (SKY) is also a hybridization molecular cytogenetic technique used to dye the different pairs of chromosomes with different colors. Chromosome-specific fluorescent-labeled probes with diverse types of fluorophores are applied. Since the number of fluorophores is limited, a combination of the colors is made. The images are analyzed by a combination of epifluorescence microscopy, charge-coupled device (CCD) imaging, and Fourier spectroscopy, which enables the analysis of all emission spectrum with a single exposure of every image points.^23^,^25^,^26^

#### Gene screening tests

These genetic tests are based on analysis of the individual gene sequence. Unlike cytogenetic tests, they are not directed at the chromosome morphology, but rather at nucleotide sequence and organization.

Allele-nonspecific and specific tests are screening assays designed to detect the presence of unknown or known mutations, respectively. These screening tests do not actually sequence nucleotides directly but detect the presence of a variation in the sequence. They can be used for population screening for a particular mutation, but are also useful for single patients, especially when evaluating a proband with an autosomal recessive disease, since the probability of detecting at least one of the two altered alleles is two times higher than finding the altered allele in a dominantly inherited disease where only one altered allele is present. Once one mutation is detected, it makes it likely that this is the gene of interest and the second mutation can be searched for using direct sequencing.

#### SSDGE or SSCP

The single-strand DNA gel electrophoresis (SSDGE) also called single-strand conformational polymorphism (SSCP) is an allele-nonspecific test. Polymerase chain reaction (PCR) is used to amplify a target DNA sequence. This is followed by denaturing the double-stranded DNA through heat and formamide (or other denaturing agents), generating single-stranded DNA molecules without altering the three-dimensional structure of the generated single strands. These molecules are submitted to gel electrophoresis under non-denaturing conditions in order to maintain their inherent spatial DNA structure. This principle is particularly important because if there is a mutation, even a single nucleotide substitution, it will potentially alter the conformation of the DNA molecule since the intra-molecular interactions will be different, therefore causing an altered molecule mobility through the gel and consequently a different molecule migration speed, which in turn will create a band corresponding to this altered molecule in a different location at the end of the gel electrophoresis when compared with a normal DNA molecule for the same fragment studied.^27^–^30^ If the result of the SSDGE is altered then direct sequencing of the PCR product can be performed in order to assess the exact mutation.

#### DGGE

The denaturing gradient gel electrophoresis (DGGE) technique is also an allele-nonspecific test and requires an initial PCR reaction that generates a large number of copies of the double-stranded DNA molecules that need to be studied. Usually, the forward primer has a 40-base GC-rich segment called “GC clamp” attached to its 5´ end to facilitate detection of mutations by the DGGE. This method has been effective for amplicons between 200 and 700 bp. The amplified dsDNA is then loaded into a perpendicular acrylamide gel containing an increasing concentration of a denaturing agent like formamide/urea for example. In order to provide this denaturing gradient, a minimum and a maximum concentration of denaturing agents must be chosen so that the gel is made with a device called gradient wheel, which will deliver the low and the high concentrations of denaturing agents in a way that the gel will be less concentrated on the top and more concentrated in the bottom. As the molecules migrate into the gel, they are increasingly submitted to higher denaturing conditions, which will gradually cause the separation of the double strands, and as a result, a speed decrease in the migration rate will occur until it practically stops moving. The timing of denaturing is directly linked to the nucleotide sequence of the dsDNA molecule. Therefore, when a mutation is present the molecule will present a different behavior under denaturing conditions and thus, a distinct speed, providing a band in a different location when compared to a normal dsDNA molecule.^31^,^32^

#### RFLPs

Restriction fragment length polymorphism (RFLP) is an allele-specific test based on the different fragment lengths of DNA generated after digestion by restriction endonucleases. The principle of the test relies on the fact that if a mutation occurs in a particular restriction site, and if the proper restriction enzyme for that site is used, there will not be any cutting, since the enzyme will not be able to recognize that site anymore due to the mutation. Therefore, in order to detect this size alteration an agarose gel electrophoresis can be performed side by side with a non-mutated DNA to show size alteration. Alternatively, a Southern-blot analysis with a site-specific probe can be done.^33^,^34^

#### Dedicated DNA microarrays

Dedicated DNA microarrays are allele-specific tests based on matrix hybridization platform reactions between hundreds or thousands of different dot-arrayed immobilized probes with a known (dedicated) mutation or single nucleotide polymorphisms (SNPs) against the patient’s amplified DNA hotspots. The detection approach is usually obtained by fluorescence of the labeled probe. The probes that can be synthetic oligonucleotides, cloned cDNAs or PCR products are immobilized as tiny dots in nonporous solid supports made mainly of glass, but alternatively of plastic or silicon allowing easy robotic automated probe microprinting delivery. These hundreds to thousands of probes are bound to the solid surface after being subjected to a chemical modification in their 3´-ends with the finality of creating a covalent ligation between the probe and the surface. Alternatively the solid surface can be chemically treated also to enhance probe ligation. These probes, after the microprinting has taken place, appear on the slide surface as a series of different pinpoint dots of DNA molecules organized in an arrayed fashion permitting a proper spatial separation between the multiple reactions that will take place simultaneously when the patient’s amplified target DNA is applied. The density of oligonucleotides per each dot on the glass surface is approximately 0.1 pmol/mm^2^, totaling around 60 billion DNA probe molecules per square millimeters. After the hybridization has taken place and the washing steps are performed, the signal emitted by the labeling must be captured and transformed into an image in order to be analyzed. Normally, the glass slide is entirely scanned and the intensities generated by each dot of the array are displayed aligned with the corresponding probe sequence so that the encountered mutation can be identified. For example, if a patient is clinically suspected of having a genetic eye disease known to be caused by a variety of mutations in one or more genes; and if screening using a dedicated array that tests for known mutations in such genes is negative, this implies, assuming a low percentage of false-negative results, that either the clinical diagnosis is wrong, or that the patient carries a novel mutation. Thus, a negative result does not exclude the presence of a mutation in the gene(s) of interest.^35^–^37^ Microarrays are in clinical use for the detection of known mutations in patients with LCA, BBS, Usher Syndrome as well as other recessive retinal dystrophies and other disorders. While they are relatively cheaper than direct sequencing methods, they offer a lower rate of detection of the responsible mutations. The earliest microarrays for the genetic testing of eye diseases were offered by Asper Ophthalmics (Tartu, Estonia).

#### Generic DNA microarrays

Generic DNA microarrays utilize the same molecular principles as the dedicated DNA microarrays. They are however based on a hybridization platform formed by a random (generic) oligonucleotide matrix, i.e. all mathematical nucleotide combinations of a probe of a certain size are oligomerized and then applied to the solid surface. In contradistinction to the dedicated DNA microarrays, copy number changes can be assessed.

#### Direct DNA sequencing

Direct DNA sequencing is based on the chain termination method first devised by Sanger. This technique is based on the existence of four deoxyribonucleoside triphosphates (dATP, dTTP, dCTP, dGTP) and DNA polymerase that will together start the synthesis of a new DNA strand after a short primer has hybridized to the template. The technique uses dideoxynucleotide triphosphates (didATP, didTTP, didCTP, didGTP), which when added to the elongation reaction forces its arrest once didNTPs lack a 3’ hydroxyl group that is necessary for the phosphodiester ligation with the next nucleotide. In the protocol, four tubes are set up, each containing a DNA template, a primer, DNA polymerase, the four dNTPs (one of which is radioactively labeled), and a single type of didNTP is added to each of the four tubes. Starting at the primer, the DNA polymerase randomly adds complementary dNTPs or didNTPs to the template. The ratio dNTPs/didNTPs is adjusted so that a didNTP is added once every 100 dNTPs. This way, each time a didNTP is incorporated, the fragment stops elongating and a distinct size of DNA molecule is generated. After replication, millions of copies of the template DNA terminated at each nucleotide are achieved. When the reactions are done, a denaturant (formamide) is added to separate the strands. Each reaction is loaded into a different polyacrilamide urea containing gel lane for electrophoresis, which is followed by X-ray exposure, and finally, manual sequencing or optical scanning reading.^38^–^43^ Although manual sequencing using the Sanger method can be used, it has been largely replaced by automated sequencing using complex apparati that sequence very large number of samples in short periods of time.

## CLINICAL PRACTICE APPLICATION OF GENETIC TESTING IN OPHTHALMOLOGY

Not long ago, knowing that a patient had the distinct phenotype of a certain genetic disease would be enough to confirm the diagnostic hypothesis. Nowadays, however, ascertaining the patient’s genotype is essential for a number of reasons. First, a precise molecular diagnosis provides the patient and his/her family important information about the disease’s nature, course, and prognosis, although genotype-phenotype correlations remain fairly limited in most instances. One example of this situation would be the patient with a mutation in the dominant retinitis pigmentosa gene such as rhodopsin or peripherin/RDS as compared with a second one with a similar phenotype early in the course of the disease, but this time due to a mutation in an X-linked retinitis pigmentosa gene. The first patient will probably have a milder clinical course than the second. A molecular diagnosis also helps differentiate genetic diseases from phenocopies caused by infectious, teratogenic or other environmental causes. It also allows precise genetic counseling and in some instances prenatal or presymptomatic diagnosis. Although gene therapy is only available for a very limited number of conditions, other preventive measures such as avoidance of vitamin A supplementation in patients with Stargardt disease and ABCA4 mutations are possible. Finally, even if the clinician is dealing with genetic diseases for which there are no significant genotype–phenotype correlations, and for which no therapy or prevention is available, it is important to make a precise genetic diagnosis for two major reasons: first, gene therapy may be imminent, and second patients generally want to know the risks of transmitting the condition to children and parents of having additional affected children. Some families have considered and utilized in vitro fertilization followed by pre-implantation genetic diagnosis to achieve disease-free pregnancy outcomes.^44^–^49^
